# Endoscopic transorbital eyelid approach for the removal of an extraconal cavernous venous malformation: Case report

**DOI:** 10.3389/fsurg.2022.954530

**Published:** 2022-07-21

**Authors:** Gianluca Lorenzo Fabozzi, Elena d’Avella, Matias Burroni, Antonio Romano, Luigi Maria Cavallo, Domenico Solari

**Affiliations:** ^1^ Department of Neurosciences and Reproductive and Dental Sciences, Division of Neurosurgery, Federico II University of Naples, Naples, Italy; ^2^ Department of Neurosurgery, Hospital Pedro de Elizalde, Buenos Aires, Argentina; ^3^ Department of Neurosciences and Reproductive and Dental Sciences, Division of Maxillofacial Surgery Unit, Federico II University of Naples, Naples, Italy

**Keywords:** cavernous venous malformation, endoscopic eyelid approach, extraconal, orbit, protosis

## Abstract

Cavernous venous malformations (CVMs) are one of the most common benign primary orbital lesions in adults and the second most frequent cause of unilateral proptosis. Extraconal location is extremely rare, representing a favorable condition as compared to intraconal, as lesions at this level often adhere to orbital muscles and optic nerve. Herein, we report the case of a 50-year-old patient, who came to our attention because of progressive painless right axial proptosis. Magnetic resonance images were consistent with an extraconal CVM, occupying the superior temporal compartment of the orbit. Successful removal of the lesion was achieved through an endoscopic transorbital eyelid approach. The present case confirms the safety and efficacy of the endoscopic transorbital eyelid approach.

## Background and importance

Cavernous venous malformations (CVMs), historically defined as orbital cavernous hemangiomas (OCHs), are the most common vascular lesions of the orbit (1). According to the International Society of Vascular Anomalies (ISSVA), CVMs are classified as low-flow non-distensible venous malformations (2). They typically present during the fourth and fifth decades of life and about 60% of cases occur in women, shedding lights on a possible interference with female sex hormones (3, 4). CVM is usually a solitary, unilateral condition, with preferential involvement of the left orbit (5–7). An extraconal location is very unusual, being 80% of the series reported within the intraconal compartment. Contrast-enhanced MR has been described as the preferred modality for detecting CVM (8): it gives information in regards to location and anatomical relationships, albeit higher diagnostic sensitivity is obtained when associated with a CT-scan (9).

Proptosis is the most common presenting sign (about 70% of cases), with an average of 5 mm at clinical presentation, and a variable degree of progression (∼2 mm per year) (6). Other symptoms include visual acuity impairment or visual field disturbances, oculomotor impairment, pressure sensation and pain. CVMs' behaviour may be very different: some remain stable for several years, while others grow more rapidly; nonetheless, spontaneous orbital haemorrhage secondary to CVM rupture is very rare (10).

Surgical management is generally required upon compression signs over optic nerve, and/or in case of disfiguring proptosis (7). Historically, surgery has been performed by means of orbitotomies, possibly associated to frontotemporal craniotomies for lesions located at the orbital lateral compartment. With the evolution of minimally invasive techniques, endoscopic approaches have progressively gained field also to address orbital lesions. As a matter of facts, the endoscopic endonasal route has been widely adopted for lesion involving the medial and inferior aspects of the orbit with excellent outcomes (11–13). Recently, the transorbital neuroendoscopic surgery (TONES) became a possible option to safely address several lesions of the superior-lateral compartment of the orbit (14).

Herein, we report a rare extraconal CVM at the superior temporal compartment of the orbit, removed through an endoscopic transorbital eyelid approach; hence, we discuss the pros and cons of this technique also in regards of the disease and anatomy dealt with.

## Clinical presentation

**Figure 1:**
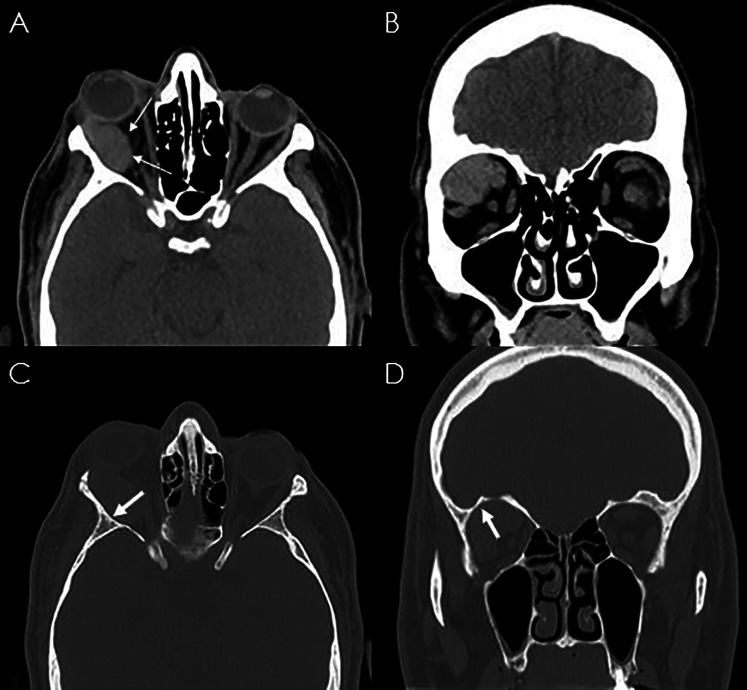
CT scan of the skull showing a right isodense well-encapsulated orbital lesion (**A**), displacing the extraocular muscles (**B**). The arrow indicates the mild bone scalloping of the orbital roof (**C,D**).

**Figure 2:**
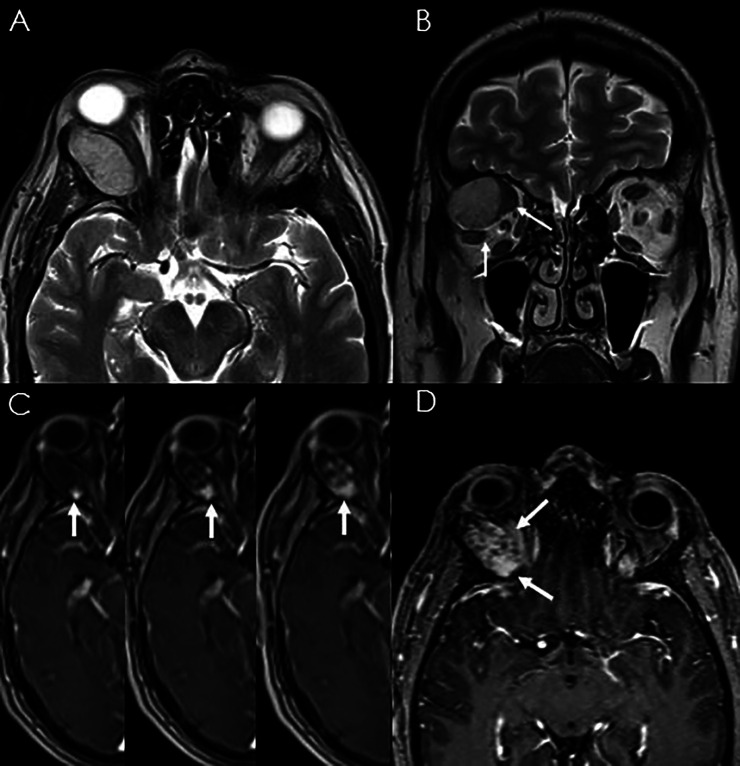
T2-weighted axial (**A**) and coronal (**B**) MRI showing the regular margin of the lesion displacing superior rectus and superior elevator palpebrae muscles medially and lateral rectus muscle inferiorly (thin arrows). Dynamic contrast-enhanced-MRI, the thick arrows indicate the progressive and centripetal enhancement after gadolinium administration (**C,D**).

A 50-year-old male patient presented with 3-month history of progressive painless right axial proptosis. His medical history was unremarkable. Ophthalmological evaluation confirmed right eye moderate proptosis, without impairment of visual functions and ocular mobility. A CT-scan showed a solid mass at the superior temporal compartment of the right orbit, measuring 32 mm × 17 mm on the axial plane and 21 mm of cranio-caudal extension. It presented regular margins and caused mild bone scalloping of the orbital roof (Figure 1). Magnetic resonance imaging (MRi) confirmed lesion at the extraconal compartment, displacing superior rectus and superior elevator palpebrae muscles medially and lateral rectus muscle inferiorly (Figures 2A,B). These muscles appeared rotated on the coronal plane, as per impingement of the Lateral Rectus – Superior Rectus band. The lacrimal gland was compressed and anteriorly displaced. Moderate right proptosis was confirmed. The lesion presented high signal on T2-weighted images with progressive centripetal contrast enhancement (Figures 2C,D). No diffusion restriction was noted. These features were consistent with a CVM.

Based on clinical and radiological findings, surgery was suggested, and an endoscopic transorbital eyelid approach was scheduled. Patient's written informed consent was obtained for scientific purposes and publication of data stripped off all identifying information. The institutional review board (IRB) at AOU Federico II (Naples, Italy) waived the need for the written consent due the retrospective nature of the study.

## Surgical approach

**Figure 3:**
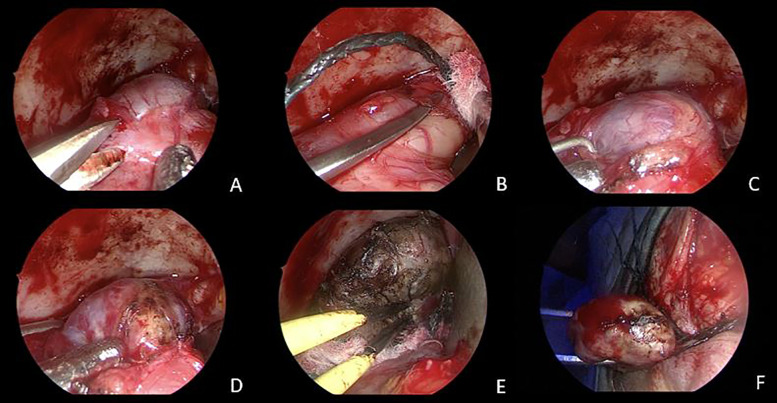
Intraoperative images. An endoscopic transorbital superior eyelid approach was performed. Sharp (**A**) and smooth (**B,C**) dissection of the CVM from the periorbita, until entire exposure of the lesion is achieved (**D**). Gentle coagulation of the capsule with bipolar forceps (**E**). *En bloc* resection of the CVM (**F**).

Surgery was performed under general anesthesia. The procedure was performed under 0° endoscopic visualization. Through a right 2.5-cm upper eyelid crease incision, the orbicularis oculi muscle was identified and dissected, reaching the superolateral border of the orbital rim. From this point over, the endoscope was used as the sole visualizing tool. The periorbita was exposed, slightly opened, and gently retracted with muscles to expose the CMV appearing as a reddish-colored, vascularized and well encapsulated mass (Figures 3A,B). With the use of dissector, curette and bipolar forceps, the lesion's capsule was gently dissected from surrounding structures and isolated from superior orbital fissure and muscles (Figure 3C); then an *en bloc* resection was successfully achieved (Figure 3D) (Video 1). Histopathological examination was consistent with CVM.

**Figure 4:**
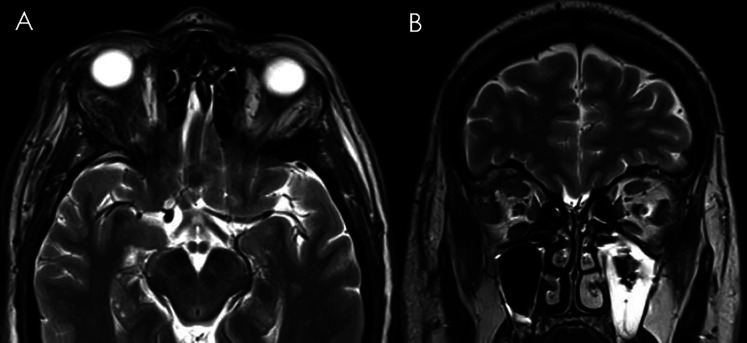
Ten days postoperative T2-weighted axial (**A**) and coronal (**B**) MRI showing complete resection of the orbital CVM with the globe's re-alignment.

The postoperative course was uneventful, and promptly proptosis reduced. Post-operative MRI confirmed the gross total removal of the lesion and ocular compression signs were resolved (Figure 4). At 3 months clinical follow-up, the clinical and cosmetic results were excellent: complete resolution of the proptosis without any morbidity nor visible scars were noted.

## Discussion

CVMs are one of the most common benign primary orbital lesions in adults (6, 7), with an incidence of 4.3% among orbital masses (15), being the most common vascular lesion of the orbit (1) and the second most frequent cause of unilateral proptosis following thyroid-related orbitopathy (16). CT and MRI scans are the primary imaging modalities used to evaluate orbital tumors and vascular lesions (9, 17). The combination of these diagnostic methods leads to an accurate diagnosis and plays a crucial role to assess relationships with the conal structures and the eventual mass effect. CVMs appear at CT-scan usually as well-circumscribed mass with homogeneous soft-tissue density and rarely signs of bone erosion. At MRI, they are found highly hyperintense on T2-weighted images and hypointense on T1; contrast enhancement increases can be patchy and heterogeneous, or it follows a more uniform pattern with centripetal extension.

Extraconal location is unusual but seems to be anatomically more favorable for surgical approach (18), as they come into direct view after the opening of the periorbita, or after minimal dissection. On the other hand, removal of intraconal CVMs requires a certain working space in between the extraocular muscles. In the present case, the pattern of displacement and rotation of extrinsic ocular muscles on MRI suggested a compression over the fibrous fascia connecting the superior margin of the lateral rectus, the lateral margins of the superior rectus and superior elevator palpebrae muscles. These findings were extremely useful in preoperative definition of the extraconal location and therefore in determining the appropriate surgical management.

Different endoscopic approaches to the orbit have been described in the literature and are typically performed using a transnasal route (11, 18–24). These techniques are safe and effective, although limited to lesions of the medial and inferior compartments of the orbit.

Regarding the superior and lateral aspects, the endoscopic eyelid approach was first described in the early 1980s for the removal of foreign bodies (25). Since then, it has gained popularity as a minimally invasive procedure for the removal of orbital tumours (26). Furthermore, the efforts in cadaveric anatomical studies have opened the way to the expand its indications: this approach became a valid alternative to expose anterior and middle cranial fossae through different transorbital corridors, for the removal of a variety of skull base lesions (27, 28). Dallan et al reported the endoscopic eyelid approach as surgical option for the removal of an intraconal CVM of the orbital apex and for the management of 9 different superior-lateral intraorbital lesions (10, 29, 30). The inner features of CVMs make their removal through endoscopic approaches favorable, being usually well encapsulated and easy to dissect from the orbital fat and surrounding intraorbital structures.

So far, transcranial routes have been preferred for the surgical management of superior-temporal orbital lesions: the lateral aspect of the orbit is usually approached *via* the minimally invasive evolutions of the fronto-orbito-zygomatic approach, such as pterional, mini-pterional approaches and supraorbital craniotomies, while lateral orbitotomies are reserved for more anteriorly located lesions (31, 32). Open craniotomies, while offering good visualization and wide surgical exposure, on the other hand, are burdened by their intrinsic invasiveness.

As compared with open transcranial techniques, endoscopic and endoscope-assisted approaches can significantly reduce morbidity and achieve comparable outcomes in selected patients (33–35). In the present case we describe the extraconal location at superior-temporal orbital area of a CVM as ideal for the surgical removal through an endoscopic transorbital eyelid approach. This technique provides an excellent exposure over the lateral and superior aspects of the orbit, including both extraconal and intraconal compartments, and allows several advantages, i.e. excellent illumination and visualization, short and direct route to the target, avoidance of bony and muscles manipulation with minimal damage to normal structures. Furthermore, low complication rates, less discomfort, better cosmetic results, and shorter hospitalization for patient are reported (10).

## Conclusions

CVMs are the most common vascular lesion of the orbit. An extraconal location is unusual but surgically favorable as the endoscopic transorbital eyelid approach might be performed for the surgical removal. This technique is effective and safe and should be considered as a solid surgical option for the management of extraconal CVM located in the lateral aspect of the orbit.

## Data Availability

The original contributions presented in the study are included in the article/Suplementary Material, further inquiries can be directed to the corresponding author/s.
